# Establishment and validation of a novel risk model based on CD8T cell marker genes to predict prognosis in thyroid cancer by integrated analysis of single-cell and bulk RNA-sequencing

**DOI:** 10.1097/MD.0000000000035192

**Published:** 2023-10-20

**Authors:** Jian Du, Cheng-Fei Song, Shu Wang, Yu-Cheng Tan, Jiang Wang

**Affiliations:** a General Surgery Department, General Hospital of Fushun Mining Bureau of Liaoning Health Industry Group, Fushun, Liaoning, China.

**Keywords:** bulk RNA, CD8T, papillary thyroid cancer, prognosis, prognosis, single-cell RNA

## Abstract

Papillary thyroid cancer (PTC) is a histological type of thyroid cancer, and CD8T is important for the immune response. The single-cell RNA data were acquired from Gene Expression Omnibus. SingleR package was used for cluster identification, and CellChat was exploited to evaluate the interaction among several cell types. Bulk RNA data obtained from the cancer genome atlas were used for determination of prognosis using Kaplan–Meier and Receiver Operating Characteristic curve. The Gene Ontology and Kyoto Encyclopedia of Genes and Genomes analysis were applied for assessment of function enrichment. The drug sensitivity was calculated in Gene Set Cancer Analysis. The regulatory network was constructed by STRING and Cytoscape. We identified 23 cell clusters and 10 cell types. Cell communication results showed CD8T cell was vital among all immune cell types. Enrichment analysis found the marker genes of CD8T cell was enriched in some signal pathways related to tumor development. Overall, FAM107B and TUBA4A were considered as hub genes and used to construct a risk model. Most immune checkpoint expressions were upregulated in tumor group. Tumor mutation burden results indicated that prognosis of PTC was not related to the mutation of hub genes. Drug sensitivity analysis showed some drugs could be effectively used for the treatment of PTC, and regulatory network identified some targets for the immunotherapy. A 2-gene model of PTC was developed based on the single-cell RNA and bulk RNA data. Besides, we found CD8T was essential for the immune response in PTC.

## 1. Introduction

Thyroid cancer (THCA) is the most common endocrine cancer, with its incidence rate consistently increasing in recent years.^[1,2]^ Based on the histology characteristics, THCA is grouped into differentiated and undifferentiated THCA.^[3]^ The patients diagnosed with well-differentiated THCA generally exhibit favorable prognoses.^[4]^

Papillary thyroid cancer (PTC), a histological type of well-differentiated THCA, represents for the majority of THCA cases.^[5]^ However, a subset of patients is diagnosed only after the tumor has progress to distant metastasis.^[6]^ In this particular scenario, some patients also demonstrate favorable prognoses when employing conventional approaches such as surgery and intensive radioactive iodine, while the majority of patients exhibit resistance to radioactive iodine, resulting in an unfavorable.^[7]^ Notably, the utilization of molecular markers has recently witnessed significant advancements in various.^[8–10]^ Consequently, the identification of novel biomarkers holds promise way for the diagnosis and treatment of PTC.

CD8T cells, also known as cytotoxic T lymphocyte, is a subtype of T lymphocytes and play vital roles in the immune response.^[11,12]^ The interaction between CD8T cells and antigen-presenting cells occurs through recognizing and binding to MHC I molecules,^[13]^ resulting in the activation and differentiation of CD8T cells.^[14]^ In general, CD8T cells differentiated into effector T cells and memory T cells.^[15]^ In the progress of immune response, effector T cells eliminate tumor cells through producing perforin and other cytotoxins,^[16]^ and memory T cells could differentiate into effector T cells when reencountering the same antigens.^[17]^ The cytotoxicity of CD8T cells is influenced by transcription factors and receptor/ligand pair.^[18,19]^ For instance, a recent study revealed that Interleukin 2 treatment has the potential to enhance the function of CD8T cells.^[20]^ In addition, the upregulation of C-X-C motif chemokine receptor 3 expression, a chemokine receptor, could promote the recognition of targets in CD8T cells.^[21]^ Moreover, previous research has demonstrated that the CD8T cells could be a target of immunotherapy.^[22]^

Thus, in this study, we identified the CD8T cell subtype using single-cell RNA (scRNA)-seq, and we explored and dissected the potential functions and mechanisms of CD8T cell genes in PTC employing bulk RNA-seq.

## 2. Methods

### 2.1. Data acquisition and processing

scRNA data of GSE191288 was acquired from the gene expression omnibus database containing 6 samples of PTC. Before the analysis of scRNA data, the cells with the number of nFeature RNA < 500 were excluded, and the cells with the number of nCount RNA < 1000 or more than 20,000 were filtered out, and the cells with mitochondria percent more than 10% were excluded. Overall, a total of 44,842 cells were chosen for further analysis. After the determination of the optimal dimension using principal component analysis, t-SNE was used for dimensionality reduction and cluster identification. Then, we identify marker genes through the Find All Markers function with min. pct = 0.25 and log2 (Foldchange) = 0.3. Finally, SingleR package was used for the cluster annotation.

The bulk RNA and copy number variants (CNV) data and corresponding clinical information of PTC were acquired from the cancer genome atlas. Besides, the immune checkpoint genes were determined based on the previous studies.^[23]^

### 2.2. The cell communication

Cell communication analysis was conducted with the scRNA-seq data by using CellChat (version 1.6.0). After the matrix of scRNA-seq and CellChat database were imported into R software, we calculated the intensity of different cell types using compute CommunProb functions. Then, we further inferred the interaction among multiple cell types through compute CommunProbPathway functions.

### 2.3. The function enrichment analysis

The marker genes of CD8T cells were identified by Find All Markers functions, and the functions of these genes were identified by the GO and KEGG enrichment analysis using the clusterProfiler R package. The minimum gene set was set to 5 and the maximum gene was set to 5000. The *P* value < .05 was considered statistically significant. The ggplot2 packages in R were used for the visualization of GO and KEGG analysis results.

### 2.4. The identification of differentially expressed genes (DEGs) and the construction of risk model

The limma package was used to determine the DEGs between 2 groups. The *P* value < .05 was set up to screen DEGs, and 78 genes were screened out in the intersection of DEGs and mark genes of CD8T cells. Besides, after the hub genes were screed out and the corresponding regression coefficients were calculated through univariate and multivariate cox analysis, we constructed the prognosis model. To assess the accuracy of the prediction, we calculated the area under the curve (AUC) for 1-, 3-, and 5-year survival using pROC package.

### 2.5. Survival analysis and tumor mutational burden (TMB)

Kaplan–Meier plotter is a common tool for assessing the effect of factors in the prognosis of cancer. The maxstat R package was used for the determination of the optimal truncation value, and the samples were divided into 2 groups according to the value. Subsequently, the survival curves were finally plotted by the survfit function of the survival package.

Then, we obtained the single-nucleotide polymorphism data from the cancer genome atlas and calculated the TMB through maftools R package.

### 2.6. Drug sensitivity analysis

We performed drug sensitivity analysis of hub genes in GSCA, which integrated over 750 small molecule drugs from genomics of drug sensitivity in cancer (GDSC) and cancer therapeutics response portal (CTRP).

### 2.7. The establishment of regulatory network

Firstly, the protein-protein interaction (PPI) network of hub genes was constructed using STRING website. The minimum required interaction score was set to 0.15, and the max number of interactors was no more than 10 interactions. Then, we predict the miRNAs and transcription factors (TFs) targeted these mRNA according to miRTarBase 8.0 and ENCODE. Besides, we reconstructed the regulatory network using Cytoscape software.

### 2.8. Statistics analysis

In this study, the statistical operation was performed in SPSS 25 (IBM Corp, Armonk, NY) and R studio software. The difference between the 2 groups was compared with an independent sample *t* test. The survival difference was determined by the log-rank test. The correlation among CNV, survival time, drug sensitivity and gene expression were calculated by the Pearson test. *P* < .05 was considered a significant difference.

## 3. Results

### 3.1. scRNA data processing and analysis

**Figure 1.**
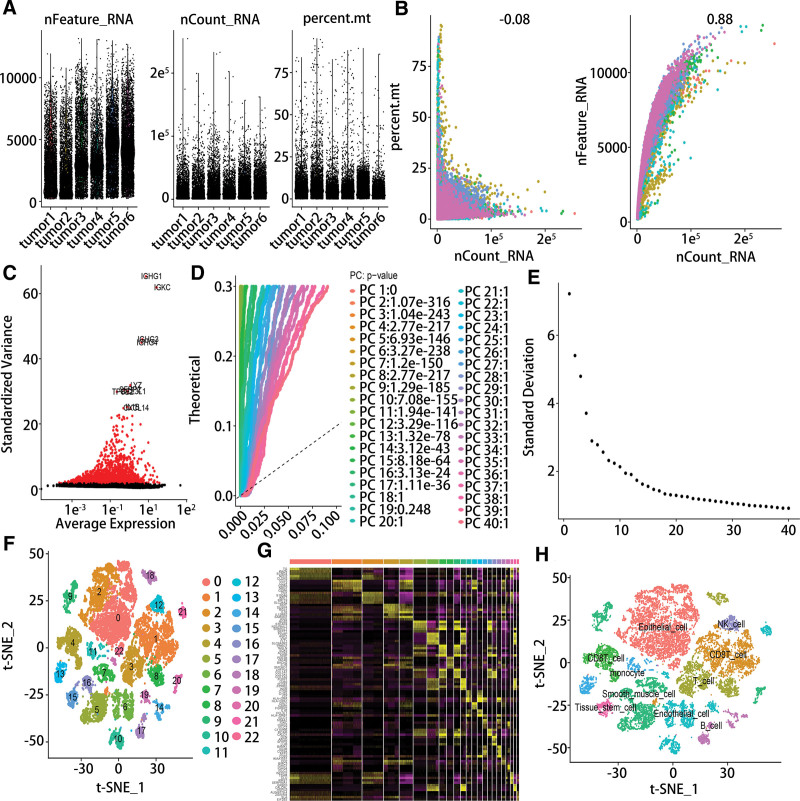
Single-cell profile of PTC. (A) The quality control of scRNA-seq (B) The correlation between nCount and mitochondria percent or nFeature. (C) The high variable genes in PTC. (D-E) The 40 PC were identified. (F) 23 cell clusters were identified using t-SNE (G) The marker genes of every cluster were visualized by heatmap. (H) 10 cell subtypes were annotated using singleR. PTC = papillary thyroid cancer, scRNA = single-cell RNA.

After the scRNA data of 6 samples were integrated, the sequencing depth, gene numbers and the percentage of mitochondria were presented in Figure 1A. As shown in Figure 1B, there was no correlation between sequencing depth and percentage of mitochondria, but there was an obviously and significantly correlation between gene numbers and sequencing depth. Totally, 8446 cells were excluded and 18,617 cells were used for further analysis after quality control. Then, we identified 2500 variable genes and the top 10 genes in the scatter diagram (Fig. 1C). Next, according to principal component analysis analysis, 17 principal components were used for the subsequent analysis (Fig. 1D–E). Furthermore, 23 clusters were classified using the t-SNE algorithm and 10 cell types were identified based on the marker genes of every cluster, including epithelial cell, smooth muscle cell, tissue stem cell, endothelial cell, monocyte, B cell, NK cell, T cell and CD8T cell (Fig. 1F–H).

### 3.2. The interaction of different cell types

**Figure 2.**
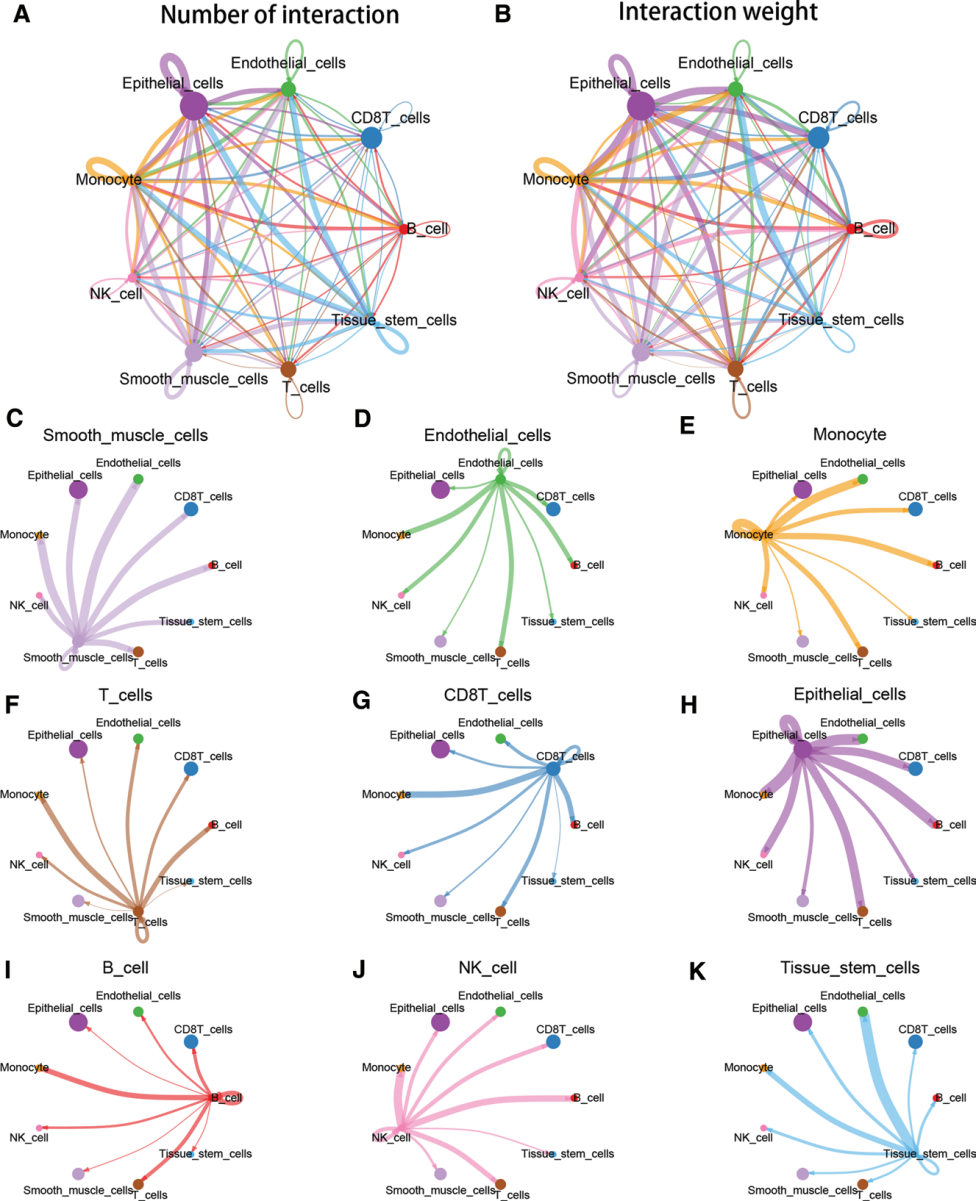
The communication of 9 cell types. (A–B) The number and weight of interaction among 9 cell types. (C–K) Interactions between every cell type and other cell types.

To better understand the connections of various types in PTC, we investigated the potential interactions among cell types using cellchatDB. From Figure 2A–B, it was obvious that epithelial cells and CD8T cells had the most and strongest interactions. The detailed interaction network between the 9 cell types was exhibited in Figure 2C–K. It could be seen that 4 immune cells containing T cells, CD8T cells, B cells and NK cells all had the strongest interaction with monocyte cells. Additionally, the receptor ligand pairs in supplement Figure S1, Supplemental Digital Content, http://links.lww.com/MD/K400.

### 3.3. The function enrichment of CD8T marker genes

Next, the functions of CD8T marker genes were determined using GO and KEGG enrichment analysis (Figure S2 A–D, Supplemental Digital Content, http://links.lww.com/MD/K401). The results showed that genes were enriched in immune system process, immune response, leukocyte activation, cell activation and T cell activation (Figure S2A, Supplemental Digital Content, http://links.lww.com/MD/K401). Besides, they mainly existed in cytosol, nuclear part, vesicle, intracellular non-membrane-bounded organelle and protein-containing complex (Figure S2B, Supplemental Digital Content, http://links.lww.com/MD/K401). In the aspect of molecular function, they were enriched in enzyme binding, enzyme regulator activity molecular function regulator, nucleic acid binding and catalytic activity (Figure S2C, Supplemental Digital Content, http://links.lww.com/MD/K401). Additionally, KEGG results revealed that the main signal pathways included T cell receptor signaling pathway, chemokine signaling pathway, NF-kappa B signaling pathway, TNF signaling pathway and Ras signaling pathway (Figure S2D, Supplemental Digital Content, http://links.lww.com/MD/K401).

### 3.4. The risk model was constructed based on differentially expressed CD8T marker genes

**Figure 3.**
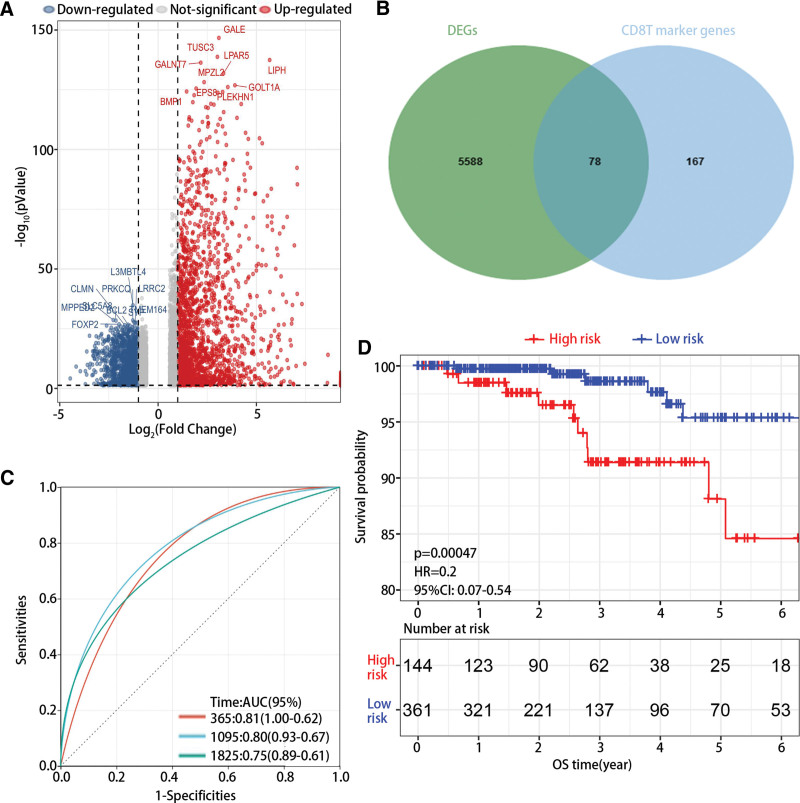
The construction and validation of risk model. (A) The differentially expressed genes were plotted in a volcano plot. (B) The intersection of DEGs and CD8T marker genes. (C) The ROC curve of risk model. (D) The survival analysis of high-risk group and low risk group in PTC. DEGs = differentially expressed genes, PTC = papillary thyroid cancer.

At first, the DEGs were screened out based on bulk RNA-seq (Fig. 3A). Then, 78 overlapped genes between DEGs and CD8T marker genes were screened out (Fig. 3B). Moreover, 2 hub genes, namely family with sequence similarity 107 (FAM107B) and tubulin alpha 4a (TUBA4A), were screened out using univariate and multivariate cox analysis (Table 1), and risk model was constructed as follow: risk score = 0.232 × exp (FAM107B) −0.124 × exp (TUBA4A). The ROC results showed the AUC for 1-,3-, and 5-year was 0.81, 0.80 and 0.75 (Fig. 3C), and Kaplan–Meier plot revealed that high risk was closely associated with poor prognosis (Fig. 3D).

**Table 1:** The univariate and multivariate cox regression analysis.

Variables	*P* value	Univariate cox analysis			*P* value	Multivariate cox analysis		
		HR	95% of HR			HR	95% of HR	
			Lower	Upper			Lower	Upper
IDI1	<.001	1.42	1.19	1.70	.067	1.280	0.98	1.67
FAM107B	.004	1.07	1.02	1.12	.001	1.351	1.13	1.61
RGS2	.004	1.06	1.02	1.10	.515	1.057	0.89	1.25
ZNF331	.005	1.12	1.03	1.22	.307	1.065	0.94	1.20
PDE4B	.005	1.34	1.10	1.65	.229	1.465	0.79	2.73
TUBA4A	.02	0.88	0.79	0.98	.018	0.867	0.77	0.98
ELL2	.02	1.12	1.02	1.24	.341	0.936	0.82	1.07
DDIT4	.02	1.04	1.00	1.07	.176	1.056	0.98	1.14
BCL2	.04	1.09	1.00	1.19	.350	0.842	0.59	1.21

BCL2 = BCL2 apoptosis regulator, DDIT4 = dna damage inducible transcript 4, ELL2 = elongation factor for RNA polymerase II 2, FAM107B = family with sequence similarity 107 member B, HR = hazard ratio, IDI1 = isopentenyl-diphosphate delta isomerase 1, PDE4B = phosphodiesterase 4B, RGS2 = regulator of g protein signaling 2, TUBA4A = tubulin alpha 4a, ZNF331 = zinc finger protein 331.

### 3.5. The expressions of immune checkpoint genes (ICGs) were significantly in low-risk group compared with high-risk group

**Figure 4.**
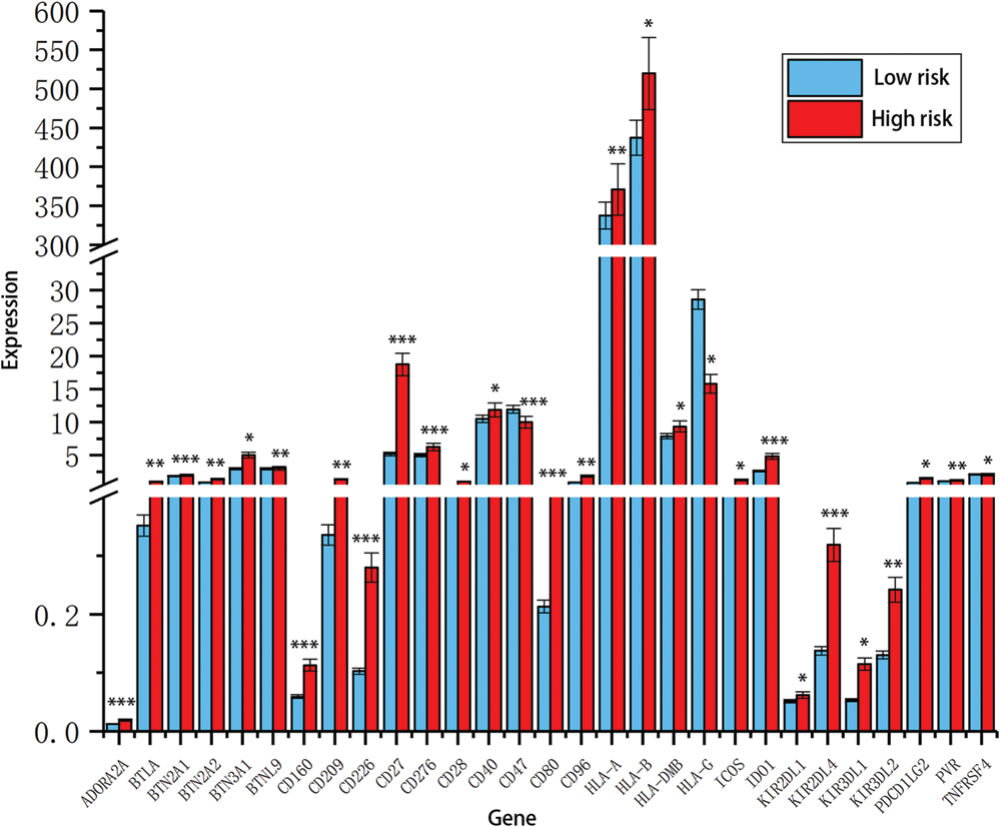
The ICGs expressions in high and low risk groups.

To investigate the potential mechanism, we first evaluated the ICGs expressions in low and high-risk groups. The results exhibited that most ICGs expressions were significantly upregulated in high-risk score group in comparison with low-risk score group, but CD47 and HLA-G were significantly downregulated in high-risk score group (Fig. 4).

### 3.6. The expression profile and survival analysis of hub genes

**Figure 5.**
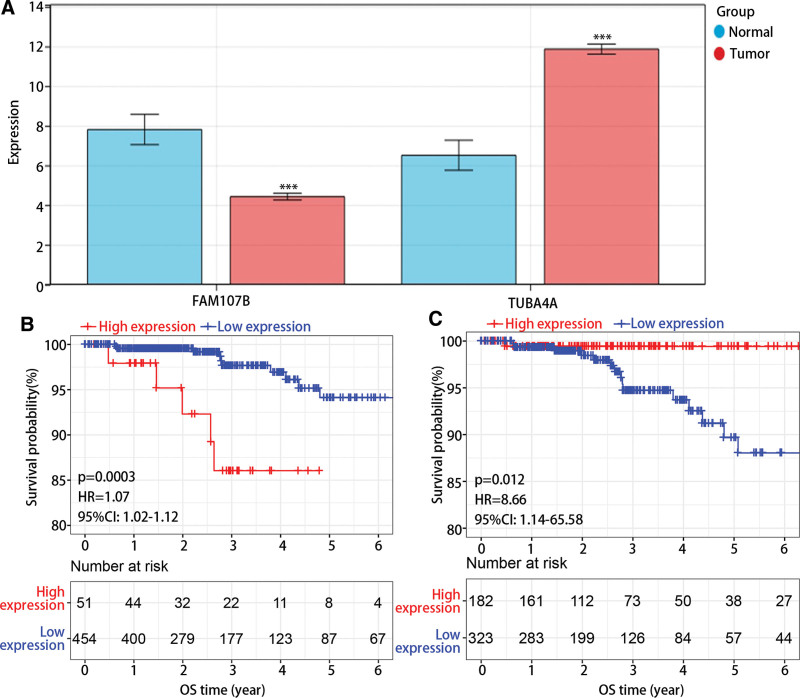
The expression and prognosis value of hub genes in PTC. (A) The expression of FAM107B and TUBA4A in normal and tumor group. (B–C) The survival analysis of (B) FAM107B and (C) TUBA4A. PTC = papillary thyroid cancer.

In addition, we found that FAM107B expression was remarkably downregulated while TUBA4A expression was remarkably upregulated in tumor group in comparison with normal group (Fig. 5A). However, survival analysis revealed that high FAM107B expression and low TUBA4A expression were associated with poor prognosis (Fig. 5B–C).

### 3.7. The landscape of TMB in PTC

**Figure 6.**
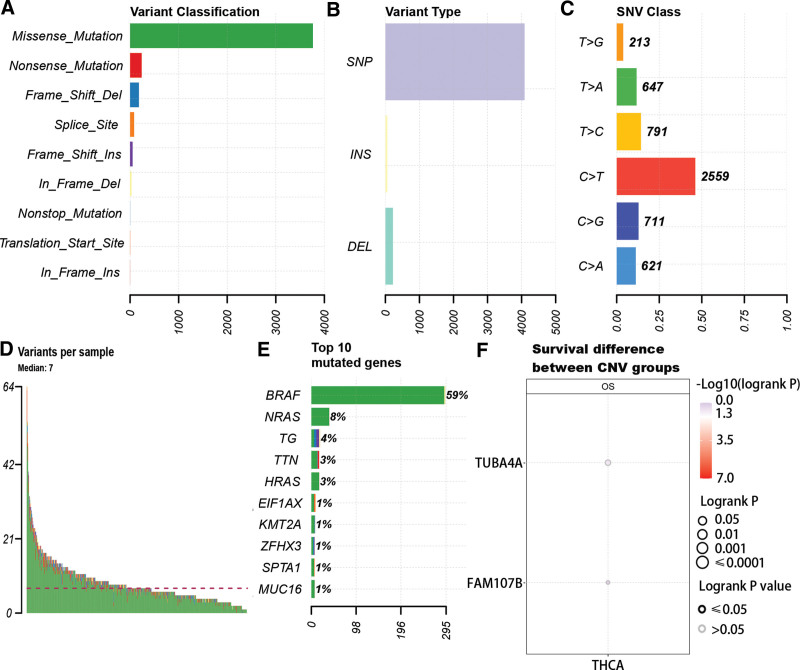
Landscape of PTC sample mutation profiles. (A–C) The mutation type in PTC. (D) The variants of every sample. (E) The top 10 mutated genes. (F) The correlation between OS and the mutation of TUBA4A and FAM107B. PTC = papillary thyroid cancer.

According to the mutation data of PTC, we found the main variant classification was missense mutation (Fig. 6A), and number of single-nucleotide polymorphism was more than insertion or deletion in PTC (Fig. 6B). In the aspect of single-nucleotide variants, C > T had the highest frequency compared with other single-nucleotide variants class (Fig. 6C). From Figure 6D–E, it was obvious that the average mutation number was 7 and most genes had a low mutation frequency except BRAF including FAM107B and TUBA4A. The further study revealed that there was no correlation between mutations of TUBA4A and FAM107B and OS of PTC (Fig. 6F).

### 3.8. The drug sensitivities were related to the expression of TUBA4A and FAM107B

**Figure 7.**
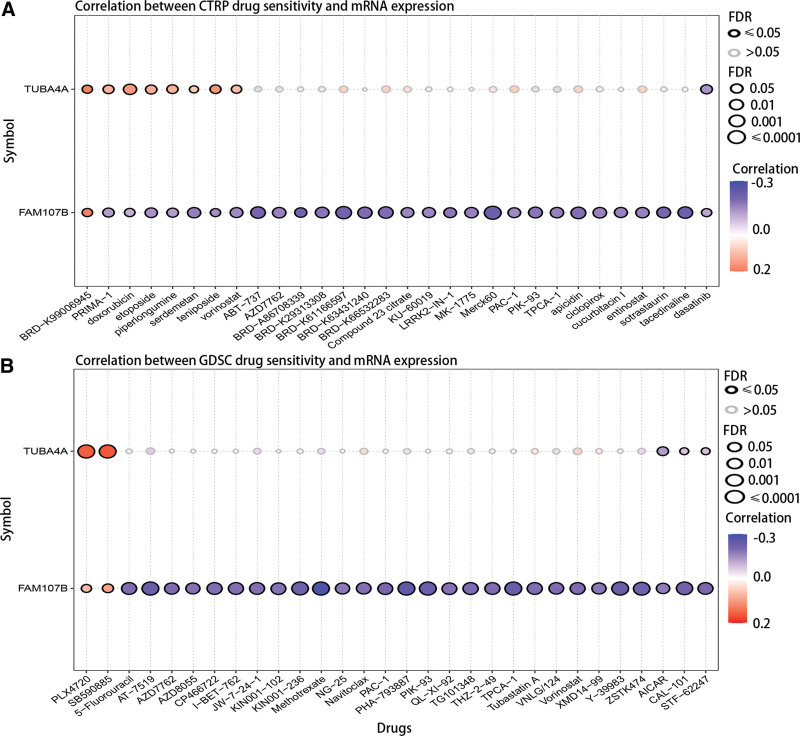
The correlation between drug sensitivities and expression of TUBA4A and FAM107B in (A) CTRP and (B) GDSC database. CTRP = cancer therapeutics response portal. GDSC = genomics of drug sensitivity in cancer.

To assess the clinical value of hub genes, we analyzed the correlation between hub genes and drug sensitivities based on CTRP and GDSC database. It could be seen that TUBA4A was positively related to some drugs but negatively related to dasatinib in CTRP database (Fig. 7A). However, FAM107B was negatively related to most drugs but positively related to BRD-K99006945 in CTRP database (Fig. 7A). In GDSC database, the results revealed that TUBA4A and FAM107B were both positively related to PLX4720 and SB590885 (Fig. 7B). Although FAM107B was negatively related to most drugs, TUBA4A was only negatively related to AICAR, CAL-101, and STF-62247 (Fig. 7B).

### 3.9. The construction of PPI and TF-miRNA-mRNA network of hub genes

**Figure 8.**
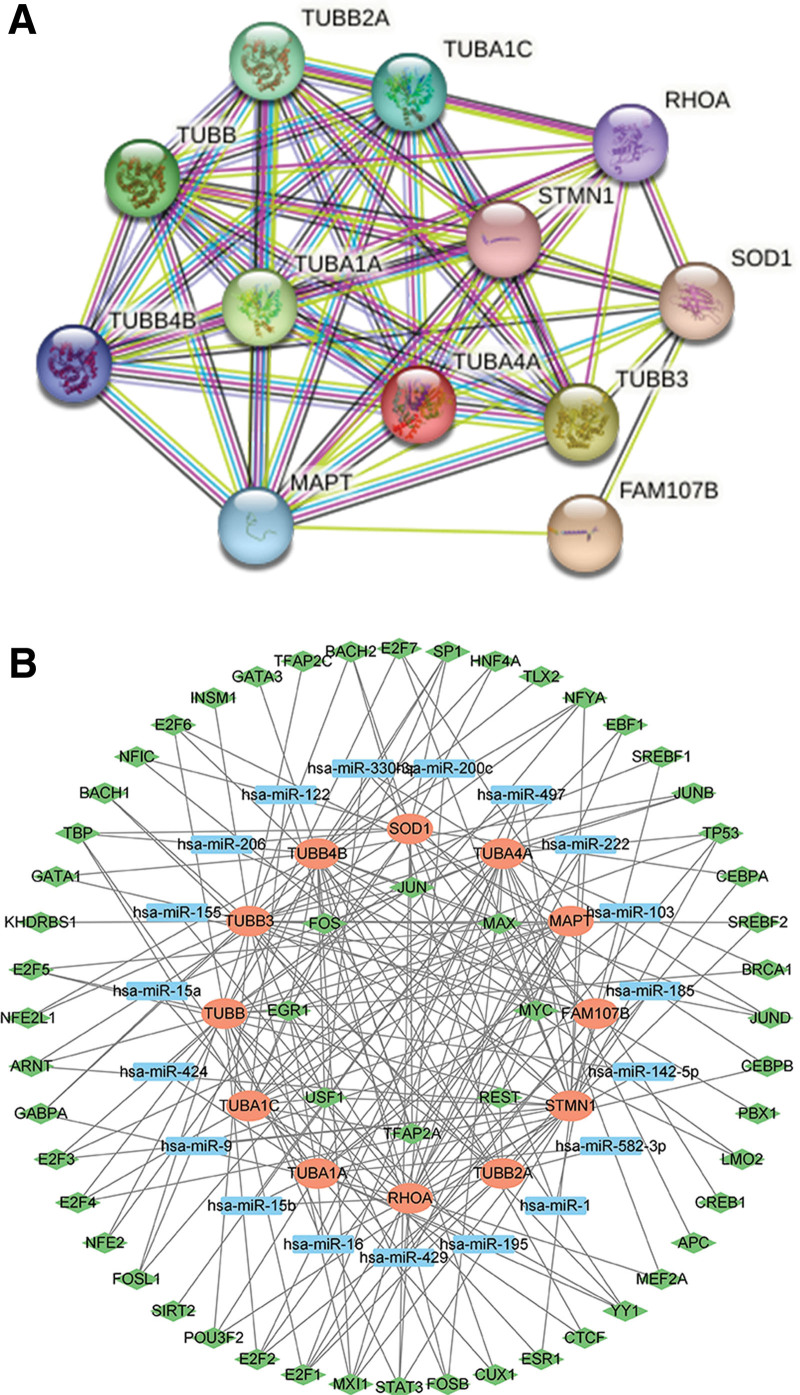
The regulatory network of TUBA4A and FAM107B. (A) The PPI network of TUBA4A and FAM107B. (B) The miRNA-TF-mRNA regulatory network of genes related to TUBA4A and FAM107B. The red plot indicated mRNA, and blue plot indicated miRNA, and the green plot indicated TF. PPI = protein-protein interaction.

Finally, we explored the potential regulatory mechanism (Fig. 8A–B). According to the STRING database, a PPI network was constructed based on FAM107B and TUBA4A, containing 12 nodes and 53 edges (Fig. 8A). Moreover, we predicted the miRNA and transcription factors of these mRNA using TarBase v8.0 and ENCODE database. After integrated analysis, a novel miRNA-TF-mRNA regulatory network was constructed, containing 12 mRNAs, 19 miRNAs and 56 TFs (Fig. 8B).

## 4. Discussion

The immune cell is an integral component of the tumor microenvironment and the intercellular communication facilitated through ligand-receptor interactions played a vital role in tumor metastasis, drug resistance and chemotherapy.^[24,25]^ Previous studies have raised that CD8T cell infiltration was associated with the results of immunotherapy.^[26]^ Furthermore, Nalio Ramos et al^[27]^ have elucidated the impact of CD8T cells on the function of macrophages in breast cancer. Additionally, Bachiller et al^[28]^ found have discovered that NK cells enhance the tumor-suppressive function of T cells, which suggested that the interaction of immune cells was meaningful for the tumor development. In our study, we identified 9 cell types including CD8T cells and determined their interaction in PTC. According to the results of intercellular communication, it was observed that CD8T cells exhibited a higher frequency of interactions with other immune cells compared to the remaining 5 immune cell types. The results also illustrated that CD8T cells predominantly engaged in interactions facilitated by the macrophage migration inhibitory factor (MIF). Some studies have identified that the application of MIF inhibitors hindered the conduction of the NF-kB signal,^[29,30]^ and overexpression of MIF facilitated the progression of osteosarcoma through activating the Ras signal pathway.^[31]^ Moreover, our analysis revealed that CD8T cell marker genes were enriched in NF-kB and the Ras signal pathways. It suggested that the MIF may promote the interaction between CD8T cells and other cells through regulating NF-kB and the Ras signal pathways. Furthermore, the upregulated expressions of most ICGs in high-risk score group indicated an enhanced immune escape of tumor cells. These results provide evidence for the crucial involvement of CD8T cells in the immune response.

FAM107 contained 2 subtypes, namely FAM107A and FAM107B. Recent studies have increasingly recognized FAM107A as a tumor suppressor, with overexpression of FAM107A inhibiting proliferation and promoting cell apoptosis.^[32,33]^ In this study, we have observed a downregulation of FAM107B in tumor samples compared, which was consistent with previous studies,^[34]^ but the low expression of FAM107B was closely related to the favorable prognosis. Conversely, we have observed a downregulated expression of TUBA4A in normal samples, which obviously correlated with a poor prognosis. These findings confirmed that FAM107B functioned as an oncogene and TUBA4A acted as cancer suppressor gene in PTC. However, it is possible that they may be not directly involved in the tumorigenesis of PTC. Besides, it had been proven that mutation in certain genes could facilitate immune escape to promote tumor development.^[35–37]^ Although extensive research has been conducted on the mutation of TUBA4A in various diseases,^[38,39]^ our findings indicate that most genes, including TUBA4A, exhibit low mutation rates in PTC. Furthermore, the CNV of FAM107B and TUBA4A was not related to the OS in PTC. Our study further reveals that the immune escape in PTC is primarily attributed to the interaction of CD8T cells rather than the mutation of CD8T related genes. According to the drug sensitivity analysis, we have identified potential therapeutic targets, namely PRIMA-1, doxorubicin, etoposide, piperilongumine, serdemetan, teniposide, and vorinostat. These targets demonstrate a positive correlation with TUBA4A and a negative correlation with FAM107B. It determined that TUBA4A and FAM107B could be targets for the therapy of PTC.

Numerous studies have elucidated the role of TF in the pathogenesis of various cancers through binding to DNA strands,^[40,41]^ while miRNA was involved in cancer development through inhibiting mRNA translation or promoting mRNA degradation.^[42]^ However, there is currently limited research on the regulatory mechanisms of TUMA4A and FAM107B. Therefore, we constructed a regulatory network through predicting the miRNA and TFs associated with hub genes. Moreover, our findings indicate a significant association between these genes and certain TFs, such as MYC and FOS, which are known to be involved in the processes of proliferation, metastasis and apoptosis in many cancer types. However, the potential mechanism of these factors in the development of PTC required further study.

## 5. Conclusion

We constructed an effective risk model of FAM107B and TUBA4A and have investigated the potential functional roles and regulatory networks of these hub genes. Our findings indicated that the interactions between CD8T cells and other cellular components play a significant role in certain cancer-related signal pathways, and the mutations of FAM107B and TUBA4A were not important factors in the development of PTC. The drug sensitivity and regulatory network revealed the optimal target of chemotherapy and immunotherapy.

## Author contributions

**Conceptualization:** Jian Du, Cheng-Fei Song.

**Data curation:** Jian Du, Cheng-Fei Song, Shu Wang.

**Formal analysis:** Jian Du, Yu-Cheng Tan, Jiang Wang.

**Investigation:** Jian Du, Shu Wang, Yu-Cheng Tan.

**Methodology:** Jian Du, Cheng-Fei Song.

**Project administration:** Yu-Cheng Tan, Jiang Wang.

**Validation:** Jian Du, Shu Wang.

**Visualization:** Shu Wang, Yu-Cheng Tan.

**Writing – original draft:** Jian Du, Cheng-Fei Song, Yu-Cheng Tan, Jiang Wang.

**Writing – review & editing:** Shu Wang, Yu-Cheng Tan, Jiang Wang.

## Supplementary Material




