# Supplementary figures and images for: Establishment and validation of a novel risk model based on CD8T cell marker genes to predict prognosis in thyroid cancer by integrated analysis of single-cell and bulk RNA-sequencing
Source: Medicine (Baltimore). 2023 Oct 20;102(42):e35192. doi: 10.1097/MD.0000000000035192 (PMC10589543; doi:10.1097/MD.0000000000035192)

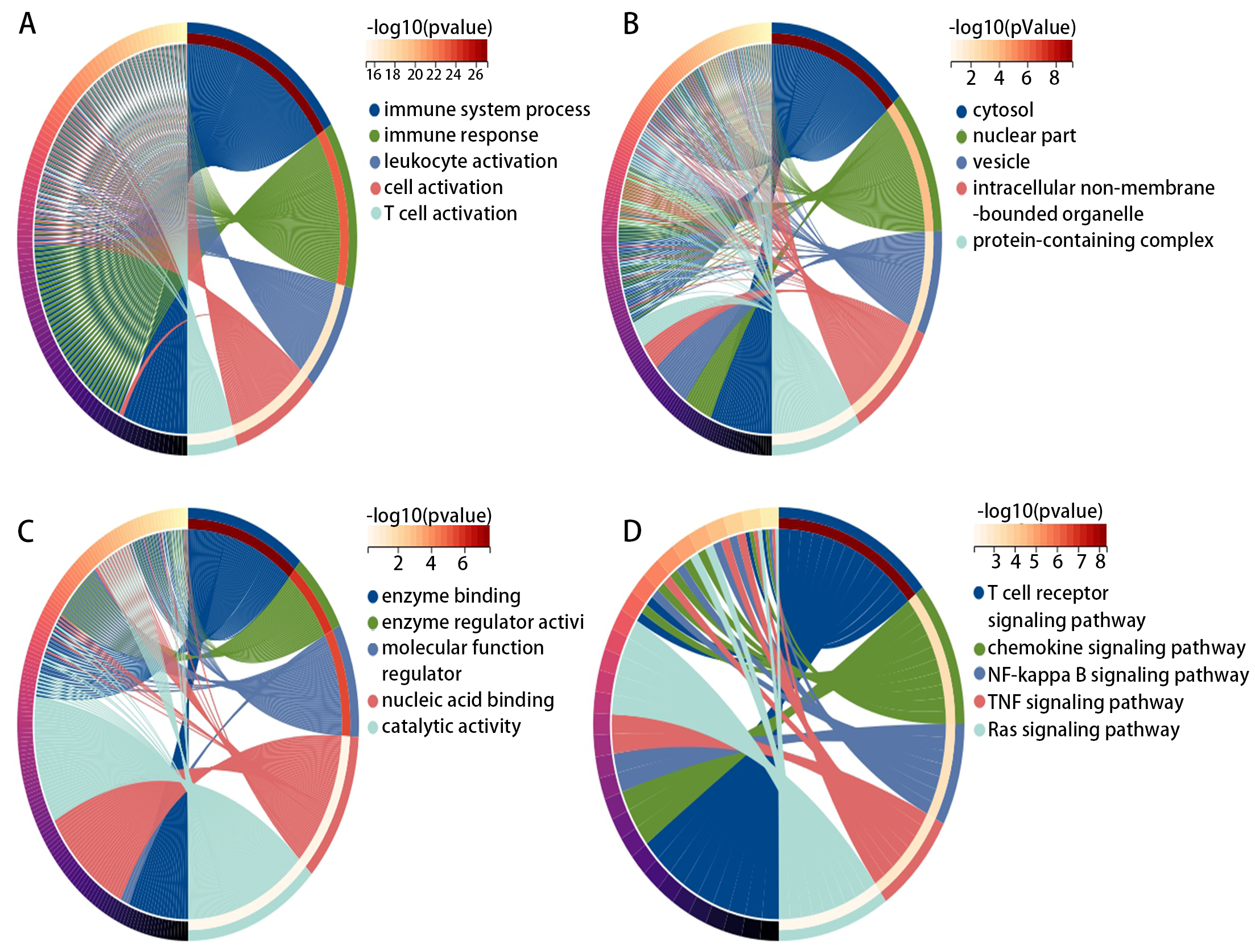

Supplement: Supplementary file 2 [file medi-102-e35192-s002.tif]
